# Can object identification difficulty be predicted based on disfluencies and eye-movements in connected speech?

**DOI:** 10.1371/journal.pone.0281589

**Published:** 2023-03-14

**Authors:** Aurélie Pistono, Robert J. Hartsuiker

**Affiliations:** Department of Experimental Psychology, Ghent University, Ghent, Belgium; University of Edinburgh, UNITED KINGDOM

## Abstract

In the current study, we asked whether delays in the earliest stages of picture naming elicit disfluency. To address this question, we used a network task, where participants describe the route taken by a marker through visually presented networks of objects. Additionally, given that disfluencies are arguably multifactorial, we combined this task with eye tracking, to be able to disentangle disfluency related to word preparation from other factors (e.g., stalling strategy). We used visual blurring, which hinders visual identification of the items and thereby slows down selection of a lexical concept. We tested the effect of this manipulation on disfluency production and visual attention. Blurriness did not lead to more disfluency on average and viewing times decreased with blurred pictures. However, multivariate pattern analyses revealed that a classifier could predict above chance, from the pattern of disfluency, whether each participant was about to name blurred or control pictures. Impeding the conceptual generation of a message therefore affected the pattern of disfluencies of each participant individually, but this pattern was not consistent from one participant to another. Additionally, some of the disfluency and eye-movement variables correlated with individual cognitive differences, in particular with inhibition.

## Introduction

**Fig 1:**
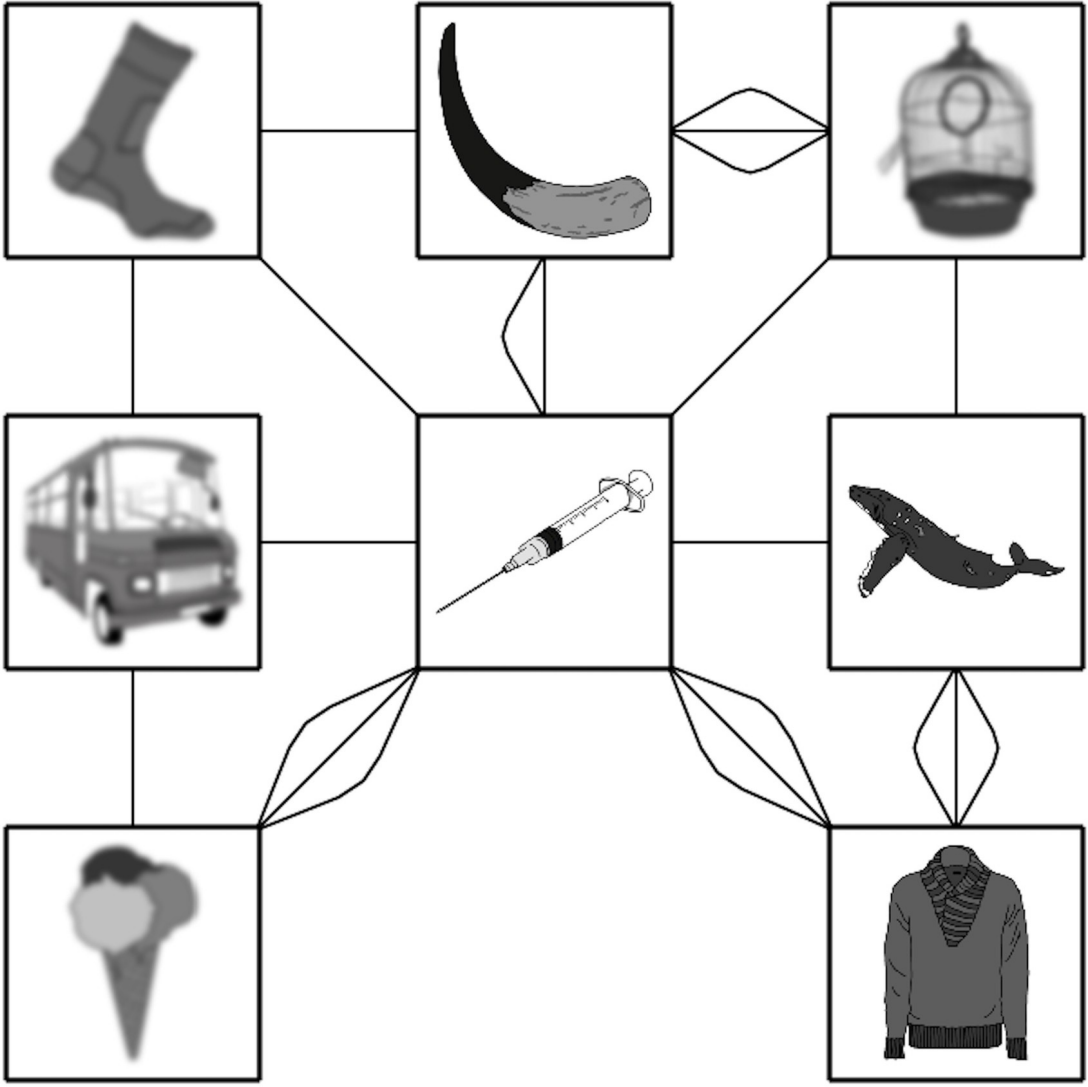
Example of a network.

The term ‘disfluency’ includes various phenomena such as filled or silent pauses, repeated words, and self-corrections. Despite the high frequency of disfluencies [1], the question remains as to why speakers are so often disfluent. Within the language system, several of the language production levels may be involved in the production of disfluencies. According to different authors [2], difficulties at distinct levels of production would lead to distinct patterns of disfluencies. However, to date, no psycholinguistic model of language production takes into account disfluencies or explains mechanistically why they occur. Influential models of language production [3,4] simulate speech errors and reaction times, but none of them simulates disfluencies. Some studies used a network task [2,5,6] to investigate precisely where disfluency comes from within the language system. In this task (Fig 1), participants describe a route taken by a point marker through a network of pictures so that a listener could fill in a blank network by listening to the description. By using databases to select pictures and control for their names [7,8], this paradigm allows for the manipulation of the items so as to create difficulties at specific production stages (e.g., lexical access difficulty, via pictures’ name agreement) while holding other stages constant (e.g., lexical frequency, phonological complexity of the pictures’ name). It has been shown, for example, that hampering the conceptual generation of a message [6], the verbal monitoring system [5] or the initial stage of lexical access [9] affected the rate of disfluencies. However, not all disfluencies are related to difficulties in speech encoding. For example, some authors stressed the role of individual differences in executive function and verbal intelligence on the fluency of speech. In particular, [10] found that repetitions were significantly related to individual differences in verbal intelligence, while silent pauses and self-corrections were marginally related to working memory measures. However, these studies did not focus on a specific level of the language production system and it is therefore not clear whether each stage relies on nonverbal cognitive abilities. Finally, some accounts suggest that some disfluencies are controlled in part top down. They consider disfluencies as a communicative act, used as words to ’signal’ delays in speech delivery to an interlocutor [11,12]. The current paper asks whether we can tease apart the multiple factors (i.e., delay in conceptual formulation of the speech plan, communicative act, or individual cognitive differences) involved in disfluency production during network descriptions.

One possible way of unravelling the mechanisms underlying disfluency production is the use of eye tracking. Indeed, speakers’ eye-movements are revealing about what their language production systems are working on. In particular, the eye-voice span before speech onset (Onset-EVS; the latency from the onset of the first fixation on a picture to the acoustic onset of its name) increases with word preparation difficulty, for example before low name agreement pictures [13], pictures with low-frequent names, or visually degraded pictures [14]. Additionally, eye-movements follow the order of picture description: from 100 to 300 ms before saying an object’s name, speakers shift their gaze to the next object to be named [15]. This lag has been argued to coincide with the end of phonological encoding. Regarding disfluencies more specifically, eye tracking indeed proved to be informative about the mechanisms underlying these phenomena, beyond difficulties related to speech encoding. In a previous study [16], we combined a network task with eye tracking, to evaluate the effects of lexical and grammatical selection on disfluencies and eye-movements. We showed that onset-EVS increased with lexical selection difficulty, which suggests that these latencies reflect the time required for word preparation during connected-speech production as well. We also revealed a strong connection between disfluencies and eye-movements: some disfluencies were associated with onset-EVS, which implies that they reflect speech encoding difficulties, while others occurred as a stalling strategy. Indeed, participants sometimes inspected other areas than the upcoming picture, while producing pauses. This suggests they made use of “strategic pauses”, consistent with the account of disfluency proposed by Clark and colleagues [11,12]. In sum, eye tracking confirmed that not all disfluencies arise from troubles within the language production system. Moreover [16], also tested whether patterns of disfluency in response to specific difficulties are robust and predictable enough to allow an algorithm (i.e., classifier) to predict which difficulty triggered them. More specifically, we tested whether the manipulated difficulty could be predicted based on the pattern of disfluency associated with it, using multivariate pattern analyses (MVPA [17]). Instead of analyzing each dependent variable individually across participants, MVPA extracts the information contained in the pattern of information available, to test whether experimental conditions can be distinguished from one another on the basis of the patterns observed, at an individual level. Applying this method to a network task indicated that lexical selection difficulty could be decoded above chance level from the pattern of disfluency and the pattern of eye-movements produced by a speaker. Thus, both patterns of disfluencies and eye-movements are sufficiently informative about this linguistic difficulty that a classifier could learn and predict the type of item a speaker was about to mention. More importantly, MVPA reinforced findings as it showed that disfluency and eye-movement patterns related to lexical selection difficulty were stable across participants.

The current study manipulates the conceptual access of object representations, to examine the pattern of disfluencies and eye-movements related to a difficulty occurring at this stage. It also tests whether problems arising at this level are associated with individual nonverbal cognitive abilities and strategies from the speakers. To do so, we used the same paradigm as [16]. In order to influence the ease of conceptual preparation of the message, we manipulated visual clarity of the target objects through visual blurring. However, contrary to previous studies using a similar manipulation [6], we first conducted a preliminary experiment that allowed us to choose the best threshold of blurriness required to elicit difficulty. We expected more prolongations before naming blurred pictures, as shown in Schnadt and Corley’s study. We also expected blurred pictures to induce longer gaze durations prior to naming in a network task, as shown during picture naming with deleted contours [14]. Furthermore, we tested whether problems associated with the conceptual preparation of the speech message could be predicted based on the pattern of disfluency and eye-movements, using MVPA. We had no hypothesis as to whether disfluency and eye-movement patterns related to visual blurring will be similar across participants.

Moreover, we expected that not all disfluencies were related to conceptualization delay. In particular, because blurriness is a salient visual difficulty, we assumed that it would encourage the use of certain strategies while performing the task (i.e., anticipatory fixations towards blurred pictures to compensate for visual processing difficulty). As we manipulate delays in the earliest stages of picture naming, speakers can identify difficulties earlier (i.e., earlier than word-form related difficulty for example), providing them with more opportunity for fluent resolution. We therefore predicted that participants with lower cognitive performance (i.e., inhibition, working memory, speed of processing, cognitive flexibility) would experience more difficulties, leading to more disfluencies and longer Onset-EVS when naming blurred pictures.

## Preliminary experiment

In order to interfere with the conceptual formulation of the message, we aimed to make identification of object images more difficult, by blurring them. We first conducted a preliminary experiment to choose the degree of degradation that was required to slow down naming while retaining high identification accuracy. Indeed, psycholinguistic studies using degraded pictures usually do not provide detailed explanations about the blurring method they used, and the degree of blurring varies considerably among studies. While Schnadt and Corley [6] used a Gaussian blur with a 1.5 pixel radius for their network tasks, several picture naming experiments used other thresholds (e.g., Gaussian blur with a radius of 6 pixels [18]; Gaussian blur with a radius of 10 pixels [19]). In our preliminary picture naming experiment, we therefore manipulated different thresholds of blurriness: Gaussian blur with a radius of 2 pixels, 4 pixels, or 6 pixels. We compared accuracy and reaction times related to these manipulations and to a control condition (no blurriness). We used a maximum threshold of 6 pixels as Laws and Hunter [18] found significantly more errors for blurred than original images in their experiment. On the contrary, we wanted to ensure that, in the network task, blurred pictures do not induce significantly more errors or “do not know” responses. The best threshold to tackle disfluency and eye-movements related to conceptual formulation difficulty will therefore be the one leading to longer reaction times in comparison to control pictures, but similar accuracy.

### Methods

#### Participants

Sixteen students of Ghent University, all native speakers of Dutch, participated. They signed an informed consent form and received 5€ for their participation. The experiment was approved by the ethical committee of the faculty of psychology and educational sciences of Ghent University.

#### Material

**Fig 2:**
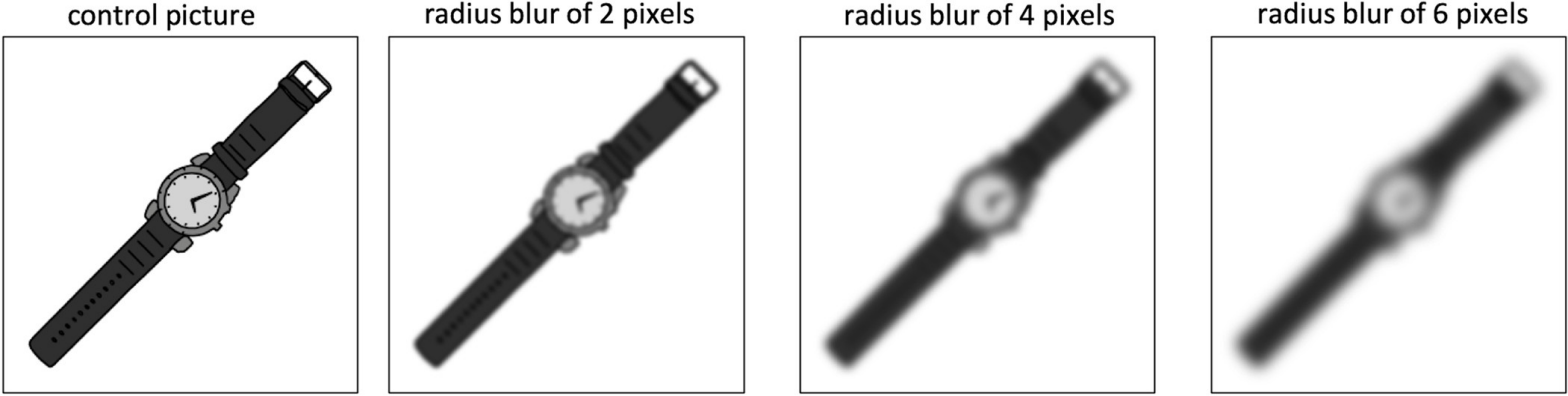
Example of each condition used for the preliminary experiment.

We selected 160 pictures from the Multipic database [7], controlled for name agreement, Age of Acquisition, and Visual complexity (see Table 1 for more information). We then applied a Gaussian filter on each picture, with a radius blur of 2 pixels, 4 pixels, or 6 pixels (Fig 2), using Photoshop software.

**Table 1:** Mean (±SD), age of acquisition (AoA), visual complexity, name agreement (H-statistic), in isolation for each set of pictures (i.e., each set was the control condition for half the participants).

	Set1	Set2	p value
AoA	6.2±1.1	6.2±1.3	0.96
Visual complexity	3±0.6	3±0.5	0.62
Name agreement (H-statistic)	0.8±0.2	0.8±0.2	0.83

#### Procedure

Using a program written in Psychopy [20] each participant named a version of all 160 pictures, under four predefined conditions to ensure all pictures were named equally often at each level of blurriness. In each version, 40 pictures were not blurred, 40 pictures had a radius blur of 2 pixels, 40 pictures had a radius blur of 4 pixels, and 40 pictures a radius blur of 6 pixels. The order of pictures was randomized across participants. Participants were instructed to name pictures as soon as they appeared on the screen.

### Data analysis

We analysed accuracy and reaction times (RTs) associated with each level of blurriness. RTs were measured from onset of the target picture until speech onset. The Chronset algorithm [21] was used to automatically extract RTs, in MATLAB. We excluded trials where participants hesitated before providing a name or produced another name before correcting themselves. Then, linear mixed effects models were performed, by means of the lme4 package in R [22] with dummy contrast coding (with the control condition as the baseline condition). Models were based on the “maximal random effects structure” approach [23] and then reduced until a further reduction would imply a significant loss in the goodness-of-fit [24].

## Results

### Descriptive

Mean reaction time was 1189 ms (± 446 ms) for control pictures; 1212 ms (± 447 ms) for 2 pixels radial blur pictures; 1233 ms (± 446 ms) for 4 pixels radial blur pictures; and 1285 ms (± 477 ms) for 6 pixels radial blur pictures. Regarding accuracy, there were, in total, 412 correct answers for control pictures (64.4%); 411 correct answers for 2 pixels radial blur pictures (64.2%); 366 correct answers for 4 pixels radial blur pictures (57.2%); and 288 for 6 pixels radial blur pictures (45%).

### Accuracy

A generalized linear mixed effect model tested for effects of blurriness on accuracy. This measure was tested with a random intercept for subjects and a random slope for blurriness over items. This resulted in a significant effect of blurriness (χ2 (1) = 46.78, p < .0001). Comparisons with the control condition indicated differences with the 6 pixels radial blur (Z = -5.72, p < .0001, β = -1.02), but not with the 4 pixels radial blur (Z = -1.74, p = .08, β = -0.28) or the 2 pixels radial blur (Z = -0.06, p = .95, β = -0.03).

### RTs

RTs were tested with a random intercept for subjects and a random slope for blurriness items. This resulted in a significant effect of blurriness (χ2 (1) = 34.92, p < .0001, η^2^ = 0.48). Comparisons showed a significant difference between the control condition and the 6 pixels radial blur (t = 5.71, p < .0001), the control condition and the 4 pixels radial blur (t = 2.74, p < .01) but the contrast with the 2 pixels radial blur was not significant (t = 0.75, p = .46).

## Discussion

The analysis of accuracy showed that the 6 pixels radial blur induced significantly more errors, similarly to [18]. This means that this threshold cannot be selected, as it hampers the visual identification of the items. Based on these findings, we considered the 4 pixels filter to be the best manipulation for the network task, as it led to longer reaction times without leading to significantly more errors. On the contrary, the 2 pixels radial blur did not differ from the control condition and is therefore not perceived as sufficiently challenging.

## Main experiment

The main experiment aimed at examining the pattern of disfluencies and eye-movements related to conceptual access difficulty (i.e., blurriness). We expected more prolongations and longer gaze durations prior to naming blurred pictures. We further expected that difficulty in conceptual preparation could be predicted based on the pattern of disfluencies and eye-movements (using MVPA). Finally, we predicted that individual nonverbal cognitive abilities are correlated with disfluency production and eye-movements related to blurred pictures.

The methods described here are similar to the one used in [16]. Data, scripts and written transcripts are made available here: https://osf.io/9yhcb/.

Transcripts, disfluency coding, and audio files are made available on FluencyBank: https://fluency.talkbank.org/access/Pistono.html.

### Methods

#### Participants

The samples have been calculated using guidelines for mixed models in designs with repeated measures, for which 1600 observations per condition are required [25]. For the current experiment, at least 20 participants needed to be included (i.e., 20 participants*((20 networks*8 images)/2 conditions) = 1600 observations per condition). Given that observations had to be excluded if the participant used the wrong name, did not produce anything, or omitted the determiner, we aimed at testing 25 participants to ensure a sufficient power. However, we had to stop the recruitment before we reached this sample size, due to restrictions on lab-based experiments as a result of the Covid-19 pandemic. Twenty bachelor students, all native speakers of Dutch, participated in the experiment in exchange for course credit (18 Females and 2 Males, mean age was 18.6±0.6 years old). They signed an informed consent form. The experiment was approved by the ethical committee of the faculty of psychology and educational sciences of Ghent University.

#### Material

We constructed 20 networks using a program written in Psychopy [20]. We used the 160 pictures from the dataset presented in the preliminary experiment. This dataset was split in two sets, matched for name agreement, age of acquisition, and visual complexity (Table 1). These parts were counterbalanced across participants, so that each part was the control condition for half the participants.

Each network consisted of eight interconnected black-and-white line pictures: four blurred (4 pixels radial blur) and four control pictures (Fig 1). Within the network, pictures were either connected by one, two, or three straight lines or curves. Lines were either horizontal, vertical, or diagonal. Curves could also be horizontal, vertical, or diagonal. The type and number of lines connecting the pictures, as well as the order and location of appearance of the 160 pictures were randomized across participants. The route through the network was indicated by a moving red dot that traversed the network in 42 seconds.

#### Apparatus

The experiment was implemented using Psychopy to display networks and record both eye tracking and speech production. Eye-movements from participants’ dominant eye were monitored with an EyeLink 1000+ system, with a sampling rate of 500 Hz. The monitor display resolution was 1921 x 1081 pixels. Pictures’ resolution was 150 x 150 pixels, subtending 2.8° of visual angle. Head movements were minimized with chin/head rests.

#### Procedure

The participants were tested individually in a quiet room. They took their place in front of a computer screen, which displayed an example network. Instructions were given to provide an accurate description of the network while staying synchronized with the dot that moved through the network. Instructions emphasized that a complete description mentioned the route of the dot, including the shape and direction of the lines or curves, and including the objects. Participants were told that their descriptions would be played to listeners who had to fill in an empty network, which only showed the position of the objects. Subsequently, three practice networks were run. The first network was described by the experimenter to illustrate the task and the next two networks were described by the participant. During this training phase, participants were already using the chin/head rest, to adapt the apparatus for the experiment if needed. During the experiment, each network was preceded by a fixation cross in the upper centre of the computer screen and started with a two seconds period for visual inspection after which the movement of the dot started. Each experiment was split in two runs of ten networks to perform a recalibration halfway through the experiment.

After the experiment, participants underwent several cognitive tests of attention, memory, and inhibition: the Trail Making Test [26], the Corsi block-tapping test [27], and the Stop-signal paradigm [28].

#### Scoring and data analysis

All productions were transcribed and scored by a native Dutch speaker. Another native Dutch speaker checked the transcriptions and scores. Disfluencies were noted for utterances related to paths, but only disfluencies preceding picture names were analysed because we manipulated properties of objects (see example provided in Table 2, only disfluencies starting from the preposition “naar” (i.e., “to”) to the object’s final name were considered). Five types of disfluency were analysed: repetitions (of a sound, syllable, word, or phrase), filled pauses, silent pauses, prolongations, and self-corrections (substitutions, additions, or deletions). Some phenomena (i.e., self-corrections) were grouped into broad categories, to ensure a sufficient amount of data within each category Silent pauses were subjectively defined by raters, as in Hartsuiker and Notebaert (2010). Example are provided in Table 2. One of the transcribers first independently transcribed and scored all networks. In a subsequent phase, a second transcriber listened to all the productions and checked the transcriptions. They disagreed on 17.8% of trials. Disagreements were solved by a third person.

**Table 2:** Definitions of each disfluency and example from a translated transcription. Disfluencies that are taken into account in the analyses (i.e., preceding pictures) are in bold.

Disfluency		Definition
**Self-correction**	Substitution [/]	When the speaker stops and resumes with a substitution for a word.
	Addition [//]	when the speaker stops and resumes with the addition of new material.
	Deletion [///]	When a speaker stops without completing an utterance and resumes with a new utterance.
	Other [////]	When the speaker stops and resumes with a grammatical or lexical error.
**Repetition** (r)		Repetitions of sounds, syllables, words or (part) phrases.
**Pause**	Silent pause (.)	When the speaker delays the speech stream by being silent.
	Filled pause (h)	When the speaker delays the speech stream by inserting a filler (e.g., uh, um)
	Prolongation (p)	When the speaker delays the speech stream by prolonging a speech sound.
**Example from a transcription, disfluencies related to pictures’ name are in bold**		
The ball starts with **the-[common gender] the-[neuter gender] [/]** piano, that there goes to the left via a straight line to **th****e** **(p)** letter, then it goes up via a small arch to the left to **the the (r)** pig (.) then (.) from the pig it goes down via a straight a small straight line [//] **t****o** **(p) the uh (h) onion**, from the onion it goes to the lef- the right [/] via a small arch down to **the (.) beard**, from the beard it goes with a small arch to the right up to **the needle (.) or sword [////],** from the **sword** it goes up via a straight line to the gorilla (.) and from (p) the gorilla it goes up via hm (h) a small arch to the right to the doll (.)		

For the analysis of eye-movements, fixation positions were categorized by object Areas of Interest (AoI) corresponding to each picture. Five variables were then considered to test the effect of blurriness on eye-movements:

- Onset-EVS: Latency (in ms) between the start of the first gaze at the picture and the onset of its name.
- Offset-EVS: Latency (in ms) between the end of the last gaze at the picture and the offset of its name.
- Number of fixations: Number of fixations occurring from the first gaze at the picture to the offset of its name.
- Number of anticipatory fixations: Number of fixations that occur on the picture before the dot traversed it.
- Number of late fixations: Number of fixations that occur on the picture after the dot traversed it.

## Results

### Descriptive

Of the total number of pictures naming attempts, 43% were excluded because the wrong target name was produced which led to 848 blurred pictures and 972 control pictures to analyse. On this final number of trials, 562 disfluencies were produced in the blurred condition and 487 in the control condition.

The proportion of disfluency produced in each condition is described below (Table 3 shows absolute values together with the percentage of pictures that had at least one disfluency of each category). Because of the high exclusion rate, some more analyses were performed on these trials, in order to control that it did not influence current findings (see Post-hoc analyses).

**Table 3:** Number of disfluency produced in control vs. blurred condition and proportion of pictures involved.

	Control condition	Blurred condition
Self-corrections	N = 74–7.3%	N = 78–8.5%
Silent pauses	N = 100–9.7%	N = 94–10.7%
Filled pauses	N = 67–6.2%	N = 46–4.8%
Prolongations	N = 99–9.8%	N = 101–12.1%

#### Disfluency

The effect of blurriness on disfluency (all phenomena together) was tested using linear mixed effects models (lme4 package in R). For the random part of the model, the maximal random effects structure [23] was included. We then chose a backward-selection heuristic, as in the preliminary experiment [24]. This resulted in a random intercept for subjects, network order, and image order, and a random slope for blurriness over subjects. There was no significant effect of blurriness (χ2 (1) = 1.49, p = .2, η^2^ = 0.01).

Generalized linear mixed effects model tested for effects of blurriness for each disfluency type (binomial) separately. There was no significant effect of blurriness when each disfluency type was analysed individually (see Appendix 1). We conducted post-hoc analyses on these results to test whether participants become highly familiar with the task, leading them to be less disfluent in the second part of the experiment. We performed t-tests to compare disfluency production in Block 1 (10 first networks) vs. Block 2 (10 last networks), which was not significant when all disfluencies were considered (t(18) = 0.76, p = 0.46), nor when considering disfluencies preceding blurred pictures (t(18) = -0.25, p = 0.81).

#### Eye-movements

Onset-EVS were log-transformed. This measure was tested with a random intercept for subjects, items, image order, and network order. This resulted in a significant effect of blurriness (χ2 (1) = 7.87, p < .01, η^2^ = 0.1), indicating longer onset-EVS before control pictures. Offset-EVS, number of fixations, and late fixations were tested with a random intercept for items, image order, and network order, and a random slope for blurriness over subjects. The number of fixations decreased with blurriness (χ2 (1) = 3.89, p < .05, η^2^ = 0.2; control pictures: 9±3; blurred pictures: 8±3) whereas offset-EVS (χ2 (1) = 0.35, p < .6, η^2^ = 0.02) and late fixations (χ2 (1) = 0.4, p < .5) did not vary with blurriness. The presence of anticipatory fixations was tested with a random intercept for subjects and items. This variable was not significantly affected by blurriness (χ2 (1) = 2.01, p < .2).

#### Post-hoc analyses

Given the high proportion of excluded trials, we conducted further analyses in which some of these trials were taken into account, in order to increase the power of the tests of blurriness on difluency and eye-movements. Excluded trials could be belong to three broad categories: semantically-related answers (i.e., trials for wich a near-synonym or semantically related answer was given); “do not know responses” and “no reponses”, which made the analysis of disfluency and eye-movements impossible; and unexpected answers (e.g., “cake” for “planet”), for which relationships with the target name were difficult to interpret. Only semantically-related answers were therefore considered for post-hoc analyses. 57.4% of these excluded trials were blurred pictures. In this data set, 30% of pictures elicited at least one disfluency. We tested the effect of blurriness, correctness, and their interaction on disfluency, onset-EVS, and number of fixations. Disfluency was tested with a random slope for blurriness and correctness over subject and random intercepts for items, network order, and image order. This effect was not significant (blurriness: (χ2 (1) = 2.63, p = 0.1, η^2^ = 0.1); correctness: (χ2 (1) = 1.36, p = 0.23, η^2^ = 0.1); blurriness* correctness: (χ2 (1) = 0.81, p = 0.37, η^2^ = 0.04)). Onset-EVS was tested with a random slope for correctness over subject, a random slope for blurriness over items, and random intercepts for network order and image order. This resulted in a significant effect of correctness (χ2 (1) = 6.03, p = .01, η^2^ = 0.21) and on correctness*blurriness (χ2 (1) = 8.54, p < .01, η^2^ = 0.1), indicating longer onset-EVS for incorrect answers and longer onset-EVS for incorrectly named control pictures. Number of fixations was tested with a random slope for correctness over subjects, a random slope for blurriness over items and random intercepts for network order and image order, indicating more fixations on incorrect items (χ2 (1) = 8.68, p < .01, η^2^ = 0.27) but no effect of blurriness (χ2 (1) = 2.79, p = .09, η^2^ = 0.1) or correctness*blurriness (χ2 (1) = 1.10, p = .30, η^2^ = 0).

#### Multivariate pattern analyses of disfluency and eye-movements

To investigate whether the conceptual manipulation could be identified based on the pattern of eye-movement or disfluency at an individual level, we performed multivariate pattern classification, using the Scikit-learn toolbox [29]. Classifiers were trained for each participant to identify whether she was about to mention a control or blurred item. We trained a linear discriminant analysis (LDA) classifier on four disfluency features (i.e., number of self-corrections, silent pauses, filled pauses, prolongations per participant and per trial) and five eye-movement features (i.e., onset-EVS, offset-EVS, number of fixations, anticipatory fixations, late fixations). In all MVPA analyses, features were normalized into Z-scores, so that the range of values for each feature was comparable across disfluency type and across participants. The Z-scoring step yielded feature distributions that were comparable across feature modality (i.e. eye-movements and disfluency production) and across participants, which allowed direct comparison of features contribution. The classification was performed in a leave-one-out cross-validation approach to ensure unbiased evaluation of classification performance: In a cross-validation fold, the classifier was trained on data from all but one trial and used on the left-out trial to predict its class membership. This procedure was repeated until each trial’s class has been predicted. Accuracy was the proportion of correctly classified trials. Classification accuracies for each analysis were compared to chance level, that is 50% for a two-class problem, using a one-tailed t-test. To determine which features played a significant role at the group level, we then performed one-sample t-tests on each feature’s contribution in the classification [30].

**Fig 3:**
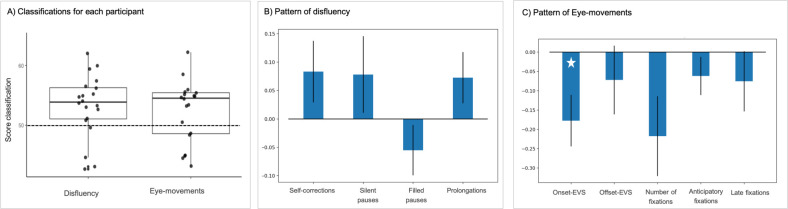
A) Classification accuracy for each participant. The dashed line indicates the level of chance. B) Contribution of each feature when classifying the pattern of disfluency. C) Contribution of each feature when classifying the pattern of eye-movements. The white star indicates significance.

Classification accuracies were significantly above chance level when analysing disfluencies (53.24% on average; t(18) = 2.77, p < .01, Fig 3). However, classifications were not above chance for each participant individually (Fig 3). Additionally, none of the four features was significant at the group level. This means that, although the pattern of disfluency could predict the type of item a participant was about to mention, this pattern was not consistent from one participant to another.

Classification accuracies were also above chance level when analysing eye-movements (53.09% on average; t(18) = 2.21, p < .05, Fig 3). In particular, onset-EVS was a significant feature for this classification (t(19) = -2.6, p < .05), meaning that onset-EVS is the most consistant feature across participant to distinguish blurred vs. control pictures. There was a trend regarding the number of fixations (t(19) = -2.05, p = .054).

#### Correlations with cognitive tests

For each condition separately, we performed Kendall correlations between cognitive tests and disfluency (i.e., proportion of each phenomenon), and eye-movements (i.e., mean onset-EVS and mean number of fixations). We chose four cognitive scores: execution time during TMT A (in seconds), execution time during TMT B-A (in seconds), Backward span, and Stop-signal reaction times (SSRT, in ms).

**Fig 4:**
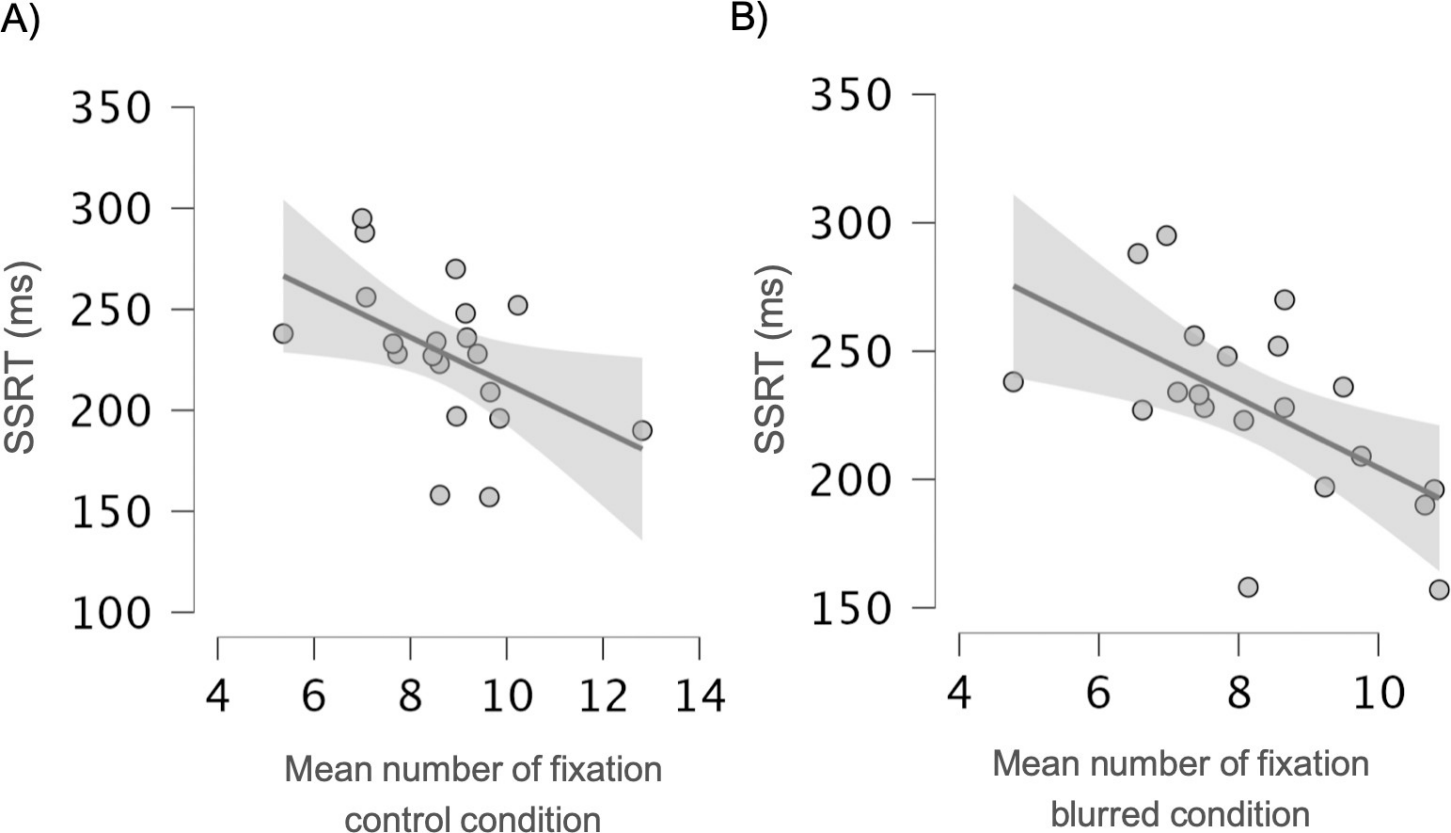
Correlations between SSRT and mean number of fixations for A) the control condition B) the blurred condition.

For the control condition, the production of disfluency was not correlated with cognitive performance. Inhibition (SSRT) was negatively correlated with the mean number of fixations (r = -0.42; p = 0.01, Fig 4), indicating more fixations on control pictures for participants with shorter reaction times and thus better inhibition capacities. Cognitive flexibility (TMT B–A) was negatively correlated with Onset-EVS, indicating more fixations on control pictures for participants who had better cognitive flexibility (r = -0.34; p = 0.04). However, this correlation was not significant after Bonferroni-Holm corrections.

For the blurred condition, inhibition (SSRT) was negatively correlated with the proportion of silent pauses (r = -0.42; p = 0.011) and with the mean number of fixations (r = -0.46; p = 0.005, Fig 4), indicating more silent pauses and fixations on blurred pictures for participants who had better inhibition capacities. On the contrary, processing speed/simple attention (TMT A) was correlated with the proportion of prolongations (r = -0.34; p = 0.04), indicating more prolongations on blurred pictures for participants with longer execution time during the TMT part A. However, this correlation was not significant after Bonferroni-Holm corrections (see Table 4 for detailed results).

**Table 4:** Correlations between cognitive performance and disfluency and eye-movements in each condition.

Blurred condition										
		Self-corrections	Silent pauses	Filled pauses	Prolonga-tions	Onset- EVS	Number fixations	TMT A	TMT B-A	Corsi
1. Self-corr.	Kendall’s Tau B	—								
	p-value	—								
2. Silent pauses	Kendall’s Tau B	0.203	—							
	p-value	0.216	—							
3. Filled pauses	Kendall’s Tau B	0.091	0.087	—						
	p-value	0.590	0.613	—						
4. Prolongations	Kendall’s Tau B	0.095	0.150	0.154	—					
	p-value	0.559	0.362	0.364	—					
5. Onset-EVS	Kendall’s Tau B	-0.090	-0.027	-0.046	0.269	—				
	p-value	0.581	0.871	0.788	0.098	—				
6. Fixations	Kendall’s Tau B	0.142	**0.400**	0.103	0.164	0.200	—			
	p-value	0.381	**0.015**	0.545	0.314	0.233	—			
7. TMT A	Kendall’s Tau B	0.005	0.238	-0.104	**0.381**	0.160	**0.364**	—		
	p-value	0.974	0.151	0.544	**0.021**	0.329	**0.027**	—		
8. TMT B-A	Kendall’s Tau B	-0.075	0.259	-0.162	0.075	-0.272	-0.037	-0.092	—	
	p-value	0.649	0.117	0.345	0.649	0.097	0.820	0.579	—	
9. Corsi	Kendall’s Tau B	-0.312	-0.183	-0.008	0.123	0.043	-0.145	0.037	-0.080	—
	p-value	0.091	0.325	0.967	0.504	0.814	0.432	0.844	0.665	—
10. SSRT	Kendall’s Tau B	-0.317	**-0.417**	-0.097	-0.063	0.069	**-0.459**	-0.311	0.000	0.152
		0.051	**0.011**	0.567	0.697	0.673	**0.005**	0.059	1.000	0.409
**Control condition**										
1. Self-corr.	Kendall’s Tau B	—								
	p-value	—								
2. Silent pauses	Kendall’s Tau B	0.275	—							
	p-value	0.091	—							
3. Filled pauses	Kendall’s Tau B	0.240	-0.059	—						
	p-value	0.143	0.720	—						
4. Prolongations	Kendall’s Tau B	0.248	0.260	0.160	—					
	p-value	0.127	0.111	0.329	—					
5. Onset-EVS	Kendall’s Tau B	0.074	-0.159	0.208	0.121	—				
	p-value	0.677	0.330	0.204	0.455	—				
6. Fixations	Kendall’s Tau B	-0.011	-0.085	0.187	-0.069	0.032	—			
	p-value	0.974	0.603	0.255	0.673	0.873	—			
7. TMT A	Kendall’s Tau B	-0.086	0.075	0.190	0.188	0.086	0.107	—		
	p-value	0.602	0.648	0.253	0.254	0.602	0.515	—		
8. TMT B-A	Kendall’s Tau B	-0.080	0.048	-0.032	0.021	**-0.336**	-0.037	-0.092	—	
	p-value	0.625	0.769	0.845	0.896	**0.040**	0.820	0.579	—	
9. Corsi	Kendall’s Tau B	-0.072	0.051	-0.132	-0.029	-0.058	-0.231	0.037	-0.080	—
	p-value	0.694	0.783	0.478	0.875	0.753	0.209	0.844	0.665	—
10. SSRT	Kendall’s Tau B	-0.185	-0.016	-0.193	-0.053	0.090	**-0.417**	-0.311	0.000	0.152
	p-value	0.256	0.922	0.241	0.745	0.581	**0.010**	0.059	1.000	0.409

#### Correlations with preliminary experiment

The current findings did not confirm our hypothesis of more disfluencies and longer onset-EVS with blurred pictures. To assess whether these variables are sensitive to picture production difficulty, we performed post-hoc Kendall’s correlations between the mean reaction time associated with each item during the preliminary experiment, and the mean onset-EVS and mean number of fixations, associated with each item during the main experiment. These correlations were performed for the 160 items, in each condition separately.

For blurred pictures, there was a positive correlation between mean reaction times during the preliminary experiment and mean number of fixations during the main experiment, but this was not significant after Bonferroni-Holm corrections (r = 0.12, p<0.05). Regarding control pictures, there was a positive correlation between mean reaction times during the preliminary experiment and mean number of fixations (r = 0.23, p<0.001) during the main experiment. There was also a positive correlation with onset-EVS (r = 0.11, p<0.05), but this was not significant after Bonferroni-Holm corrections.

## Discussion

This study showed that blurred pictures did not elicit more disfluency than control pictures overall during a network task. Additionally, time spent on pictures was shorter for blurred pictures than for normal pictures, contrary to what was expected. However, multivariate pattern analyses were able to predict above chance whether participants were about to name a blurred or non-blurred picture based on their pattern of disfluency or eye-movements associated with it. Some of the disfluency and eye-movement variables correlated with individual cognitive measures. In the next three sections, we discuss the results for the disfluencies, eye-movements, and the individual difference measures.

### Disfluency

Surprisingly, impeding conceptual access of object representations did not elicit more disfluency. However, the rate of disfluency was overall quite substantial (36% of trials had at least one disfluency) compared to studies manipulating lexical selection (e.g., [9,16]. It is therefore possible that, because the manipulation was visually salient, the complexity of the entire task increased, leading to more disfluency in both conditions. Indeed, making the identification of half the items more difficult might have hampered the monitoring system (i.e., poor error detection and correction while staying synchronized with the pace of the dot) throughout the whole task, similarly to time pressure [5] or divided attention [31].

The present results partly differ from those of Schnadt and Corley [6], who found an effect of blurriness on prolongations. However, their method was quite different: they chose a different threshold of blurriness (1.5 pixel radial blur), a different set of pictures [32], and performed different analyses (ANOVAs). Additionally, Schnadt and Corley’s study was conducted among English speakers. This could contribute to different results, as previous work showed a different pattern of hesitations in English, Dutch, and German [33]. More particularly, and as mentioned above, Schnadt and Corley found a higher proportion of prolongations for blurred pictures. This can be due to the fact that English, unlike Dutch, does not have grammatical genders marked on determiners. Participants can therefore prolongate the determiner “a” or “the” to stall for time while finding the correct name. On the contrary, Dutch speakers can not use such strategies, which might partly explain why they used different types of disfluency when facing blurred pictures (as shown with MVPA). Note however that Schnadt and Corley found significantly fewer disfluencies in their experiment using blurriness as compared to another experiment in which they manipulated name agreement and lexical frequency. According to them, this could be taken as evidence that speakers can identify conceptual difficulties earlier, providing them with more opportunity for fluent resolution.

Multivariate pattern analyses yielded complementary findings. They revealed that the classifier could predict, from the pattern of disfluency, whether each participant was about to name blurred or control pictures. This means that disfluencies were sufficiently informative about the linguistic difficulty of an item that a classifier can learn and predict the type of item a speaker is about to name better than chance. In other words, impeding the conceptual generation of a message affected the pattern of disfluencies of each participant individually. However, none of the four features (i.e., disfluency type) was affected in a consistent way across participants. This means that, although the pattern of disfluency could predict the type of item a participant was about to mention, this pattern differs from one participant to another. This finding explains why linear mixed models did not reveal significant differences: delays in conceptual processing manifest themsleves differently from one participant to another. Correlations with cognitive performance provided insights into these individual differences, as detailed in a following section.

### Eye-movements

Contrary to what was expected, the allocation of visual attention did not increase with our blurring manipulation. First, blurred pictures did not induce more anticipatory fixations, contrary to our hypothesis. However, this experiment induced more anticipatory and late fixations in general, compared to our previous experiment that manipulated lexical and grammatical selection difficulty [16]. This supports the idea that the blurring manipulation influenced eye-movement patterns across the board and not only on the manipulated pictures. Second, control pictures induced longer onset-EVS and more fixations than blurred pictures. These results differ from Meyer and colleagues [14], who found that degrading pictures significantly affected the mean naming latencies and the mean time spent looking at the objects during a picture naming task. These differences may be due to differences in task and stimuli. Indeed, during real-world scene perception, viewers usually concentrate their fixations on interesting and informative regions, while empty, uniform, and uninformative scene regions are often not fixated [34]. It is possible that a dynamic description task induced findings that are similar to real-world processing, where control pictures required more attention and gaze control than blurred pictures.

Multivariate pattern analyses reinforced findings from linear mixed models: classification accuracies were above chance level when analysing eye-movements, and onset-EVS and number of fixations were the most important features for these classifications. The pattern of eye-movements was therefore more consistent across participants than the pattern of disfluency, indicating more time spent on control pictures. This result suggests that the differences we observed reveal common underlying cognitive mechanisms that are consistent across participants. The pattern of eye-movements, and the number of fixations in particular, has proven to be highly discriminating when classifying different tasks. Using similar multivariate analyses, Kardan and colleagues [35], showed that aesthetic preference induced more fixations than scene memorization, and scene memorization induced more fixations than visual search. The current experiment shows that, within a same task, a classification algorithm can also be successful in predicting the type of pictures (i.e., blurred vs. non-blurred) being processed, based on eye-movement data.

Taken together, these findings are quite surprising, especially given that blurred pictures elicited longer reactions times in the preliminary experiment. That is why we performed correlations between mean items’ RTs during the preliminary experiment and mean items’ onset-EVS and number of fixations. By doing so, we revealed an “item effect”: items that induced longer reaction times during the preliminary experiment tended to induce longer onset-EVS or more fixations during the main experiment, in both conditions. The discrepancy between mean RTs for blurred pictures in the preliminary experiment and mean onset-EVS for these pictures in the experiment shows that the network task is not comparable with a naming task when examining conceptual difficulty. Furthermore, the current results might be influenced by the threshold we used for blurred pictures. During a network task, participants have to build complete sentences while staying synchronized with the pace of the dot, and while processing several pictures displayed on the screen. This task is therefore more complex but more importantly, the pictures displayed during the network task are slightly smaller than the ones displayed in the single picture naming task. It is therefore possible that the threshold we used became too difficult in this experimental setting. This might help explain why the current results differ from those of Schnadt and Corley -who used a threshold of 1.5 pixel radial blur- and Meyer and colleagues -who used a single picture naming task.

### Correlations with cognitive performance

Correlations with cognitive performance provided insights into the relationship between disfluency and eye-movements. Indeed, they revealed a positive correlation between the number of fixations, silent pauses, and inhibition. In other words, participants with better inhibition spent more time examining the upcoming picture, rather than inspecting other areas of interest (in both conditions), which is coherent with literature showing the existence of an inhibitory mechanism over visual attentional control [36,37]. Additionally, participants with better inhibition produced more more silent pauses in the blurred condition. The use of silent pauses as a marker of better cognitive performance during connected-speech production has previously been shown in several types of narratives [38,39]. In a sentence repetition task however, the length of silent pauses seems to be correlated with inhibition while the frequency of these phenomena is not [10]. Correlations between cognitive performance and disfluencies (e.g., between speed of processing and prolongations) were not significant after correction for multiple comparisons. These results would suggest that silent pauses and prolongations reflect different mechanisms, but future work, based on a larger sample of participants, is required to determine more thoroughly the influence of cognitive differences on disfluency production.

### Limitations and future directions

This study has several limitations. First, the sample size is rather small given that many trials were excluded due to bad identification of the object to be named, and given that it was not possible to test more participants due to sanitary measures. Indeed, a post-hoc power analysis indicated that the study is underpowered (i.e., to identify the sample size that would be required to get a power >.80, we ran simulations using the simr package [40]. Based on these simulations, 34 participants would be required). However, the absence of differences regarding disfluency production will probably be similar with more trials, as suggested by descriptive data, post-hoc analyses and MVPA. First, the number of disfluencies produced in total is quite similar for each condition, as shown in Table 3, which is quite surprising. These descriptive results are strengthened by post-hoc analyses, in which we showed that disfluencies did not increase for blurred pictures when more trials are included (i.e., when taking into account trials for which a semantically related answer was provided, such as “lion” for “tiger”). MVPA provided some insight on this lack of significance. Indeed, it showed that there is no consistency across participants, meaning that the type of disfluency produced for each condition was not similar from one participant to another. Having more participants will surely reinforce current conclusions since accuracy scores will be less affected by outliers (i.e., by participants for which classification was below chance). Another way to reinforce current findings would be to compare a language without gender marking, such as English, and Dutch speakers on this task. Indeed, this will test whether participants are more likely to use a similar strategy (i.e., producing prolongations) in a language in which determiners are not marked for gender. Further work is therefore required to replicate the current findings, and several adjustments can be made to the current paradigm. For example, other manipulations could be considered to tackle conceptual access. Indeed, blurriness may affect other processes (e.g. visual attention, similarity-based interference, etc) than conceptual formulation. Future work could use pictures that are ambiguous in what concept they denote to impede conceptual access without globally changing the properties of the visual display (i.e., an object shaped like another object). Future studies could also limit the information made available to the speaker (i.e., each picture appearing before the dot traverses it), to limit interference with visual control.

## Conclusions

Disfluency and eye-movements seem quite independent in this experiment, contrary to what has been shown regarding pictures’ name agreement. Many studies have shown that the manipulation of variables that affect object naming latencies also affect looking times in a similar way (e.g., lexical frequency, name agreement, etc.). On the contrary, in the present network task experiment, eye-movements were not linked with naming difficulties. Nevertheless, MVPA of disfluency patterns showed that conceptual difficulty manifests itself differently from one participant to another. These findings therefore point to a need for current models of language production to capture inter-individual variability. Altogether, the current findings open new directions for the use of MVPA to study the language production system.

## Supporting information

S1 File (DOCX)
