# Supplementary material for: Can object identification difficulty be predicted based on disfluencies and eye-movements in connected speech?
Source: PLoS One. 2023 Mar 14;18(3):e0281589. doi: 10.1371/journal.pone.0281589 (PMC10013892; doi:10.1371/journal.pone.0281589)
Supplement: S1 File — (DOCX) [file pone.0281589.s001.docx]

Appendix 1 - Non-significant results

| Variable | Random structure | Results |
| --- | --- | --- |
| Main analysis | | |
| Silent pauses | random intercept for item, subject, network order | (χ^2^ (1)= 0.67, p=0.41) |
| Filled pauses | random intercept for item and subject | (χ^2^ (1)= 2.6, p=0.11) |
| Self-corrections | random intercept for item and subject | (χ^2^ (1)= 1.35, p=0.24) |
| Prolongations | Random slope for blurriness over items, random intercept for subject, network order and image order | (χ^2^ (1)= 0.12, p=0.73) |
| Excluded trials | | |
| Silent pauses | random slope for blurriness over subjects, random intercept for item, network order, image order | (χ^2^ (1)= 0.3, p=0.58) |
| Filled pauses | random intercept for item and subject | (χ^2^ (1)= 0.006, p=0.94) |
| Self-corrections | random intercept for item, subject, and network order | (χ^2^ (1)= 2.41, p=0.12) |
| Prolongations | random intercept for subject and item | (χ^2^ (1)= 0.51, p=0.47) |
